# Insecticide resistance patterns in Uganda and the effect of indoor residual spraying with bendiocarb on *kdr* L1014S frequencies in *Anopheles gambiae* s.s.

**DOI:** 10.1186/s12936-017-1799-7

**Published:** 2017-04-20

**Authors:** Tarekegn A. Abeku, Michelle E. H. Helinski, Matthew J. Kirby, James Ssekitooleko, Chris Bass, Irene Kyomuhangi, Michael Okia, Godfrey Magumba, Sylvia R. Meek

**Affiliations:** 1 Malaria Consortium, Development House, 56-64 Leonard Street, London, EC2A 4LT UK; 2 Malaria Consortium Uganda, Plot 25, Upper Naguru East Road, Naguru, Kampala, Uganda; 3 London School of Hygiene & Tropical Medicine, London, WC1E 7HT UK; 4 University of Exeter, Penryn Campus, Treliever Road, Penryn, TR10 9FE UK; 5 Rothamsted Research, Harpenden, Hertfordshire AL5 2JQ UK; 6 National Malaria Control Programme, Ministry of Health, Kampala, Uganda; 7 Uganda IRS Project Phase II/Abt Associates Inc., Kampala, Uganda

**Keywords:** Malaria, *Anopheles gambiae*, Pyrethroids, *kdr*, Resistance, Bendiocarb, Insecticide-treated nets, Indoor residual spraying

## Abstract

**Background:**

Resistance of malaria vectors to pyrethroid insecticides has been attributed to selection pressure from long-lasting insecticidal nets (LLINs), indoor residual spraying (IRS), and the use of chemicals in agriculture. The use of different classes of insecticides in combination or by rotation has been recommended for resistance management. The aim of this study was to understand the role of IRS with a carbamate insecticide in management of pyrethroid resistance.

**Methods:**

*Anopheles* mosquitoes were collected from multiple sites in nine districts of Uganda (up to five sites per district). Three districts had been sprayed with bendiocarb. Phenotypic resistance was determined using standard susceptibility tests. Molecular assays were used to determine the frequency of resistance mutations. The *kdr* L1014S homozygote frequency in *Anopheles gambiae* s.s. was used as the outcome measure to test the effects of various factors using a logistic regression model. Bendiocarb coverage, annual rainfall, altitude, mosquito collection method, LLIN use, LLINs distributed in the previous 5 years, household use of agricultural pesticides, and malaria prevalence in children 2–9 years old were entered as explanatory variables.

**Results:**

Tests with pyrethroid insecticides showed resistance and suspected resistance levels in all districts except Apac (a sprayed district). Bendiocarb resistance was not detected in sprayed sites, but was confirmed in one unsprayed site (Soroti). *Anopheles gambiae* s.s. collected from areas sprayed with bendiocarb had significantly less *kdr* homozygosity than those collected from unsprayed areas. Mosquitoes collected indoors as adults had significantly higher frequency of *kdr* homozygotes than mosquitoes collected as larvae, possibly indicating selective sampling of resistant adults, presumably due to exposure to insecticides inside houses that would disproportionately affect susceptible mosquitoes. The effect of LLIN use on *kdr* homozygosity was significantly modified by annual rainfall. In areas receiving high rainfall, LLIN use was associated with increased *kdr* homozygosity and this association weakened as rainfall decreased, indicating more frequency of exposure to pyrethroids in relatively wet areas with high vector density.

**Conclusion:**

This study suggests that using a carbamate insecticide for IRS in areas with high levels of pyrethroid resistance may reduce *kdr* frequencies in *An. gambiae* s.s.

## Background

There has been a massive scale-up of malaria vector control interventions in sub-Saharan Africa in the past 15 years, mainly through universal coverage of long-lasting insecticidal nets (LLINs) and targeted indoor residual spraying (IRS) [1]. Pyrethroids are the only class of insecticides used in LLINs currently recommended by the World Health Organization (WHO).

Pyrethroid resistance of malaria vectors is widespread in Africa [2] and resistance to other classes of insecticides has been documented from numerous countries [3–7]. Globally, 60 countries reported resistance to at least one insecticide out of 73 malaria-endemic countries that provided data, and resistance to pyrethroids was the most commonly reported [1]. Increased resistance has been attributed to selection pressure from the scale-up of LLINs and IRS [2] and use of similar classes of insecticides in agriculture [8], although the relative contribution of these mechanisms varies by area [9].

The impact of resistance to pyrethroids on the effectiveness of LLINs is not well understood. A study in Malawi indicated a protective effect of LLINs in an area where *Anopheles funestus* showed low to moderate resistance against pyrethroids [10], and similar results were observed in Benin where pyrethroid-resistant *Anopheles gambiae* were the main vector [11]. A meta-analysis suggested that insecticide-treated nets (ITNs) continue to have an effect on entomological outcomes regardless of resistance [12]. An observational prospective study in areas with varying levels of pyrethroid resistance across five countries (Benin, Cameroon, India, Kenya, Sudan) did not find an association between malaria disease burden and levels of resistance, and showed that ITNs remained effective despite the increasing resistance [13]. The study also showed that development of pyrethroid resistance was slower in locations where LLINs were used in combination with IRS with a non-pyrethroid than in areas with LLINs alone.

However, other studies showed that pyrethroid resistance is a major threat to malaria control efforts [2, 14]. It is likely that over-reliance on one class of insecticides will compromise the success of malaria control in the long term. To maintain the effectiveness of current control interventions, managing insecticide resistance is of critical importance [15]. Combination of interventions using different insecticide classes has been recommended as one of the main insecticide resistance management strategies for malaria control, as part of WHO’s Global Plan for Insecticide Resistance Management [15]. Although there are a number of recommended strategies for resistance management in IRS, combining IRS and LLINs is the only option currently available for LLINs.

This study was conducted primarily to evaluate the role of using a carbamate insecticide for IRS in the management of pyrethroid resistance in *An. gambiae* s.s. in Uganda. Variations in phenotypic resistance and frequencies of knock-down resistance (*kdr*) genotypes were studied in relation to intensity of use of insecticides in public health and agriculture. The specific aim of the research in relation to this paper was to understand the effect of bendiocarb spraying on the frequency of *kdr* L1014S homozygotes (RR) (*kdr* homozygosity) while controlling for the effect of other factors that potentially influence development and spread of resistance.

## Methods

### Study sites

Forty-five health centres in nine districts in Uganda were selected using a multi-stage sampling procedure. Districts that existed since 2001 with high malaria endemicity were included in the sampling frame. The districts were then stratified into three groups as follows: (1) Group A: districts that had undergone several rounds of IRS with various insecticides and where more than 1.5 LLINs per household had been distributed during 2008–2010; (2) Group B: districts where more than 1.5 LLIN per household had been distributed during 2008–2010 but no IRS had taken place; and, (3) Group C: districts that had not received IRS or LLINs as part of a large campaign.

Three districts were selected randomly from each group as follows: Group A: Apac, Gulu and Pader; Group B: Kayunga, Kiboga and Mbale; and, Group C: Bugiri, Mayuge and Soroti. Among the Group C districts, LLINs were distributed in Mayuge and Bugiri in September 2012 as part of a national campaign, which significantly increased the coverage just as the entomological surveys were being conducted.

**Fig. 1:**
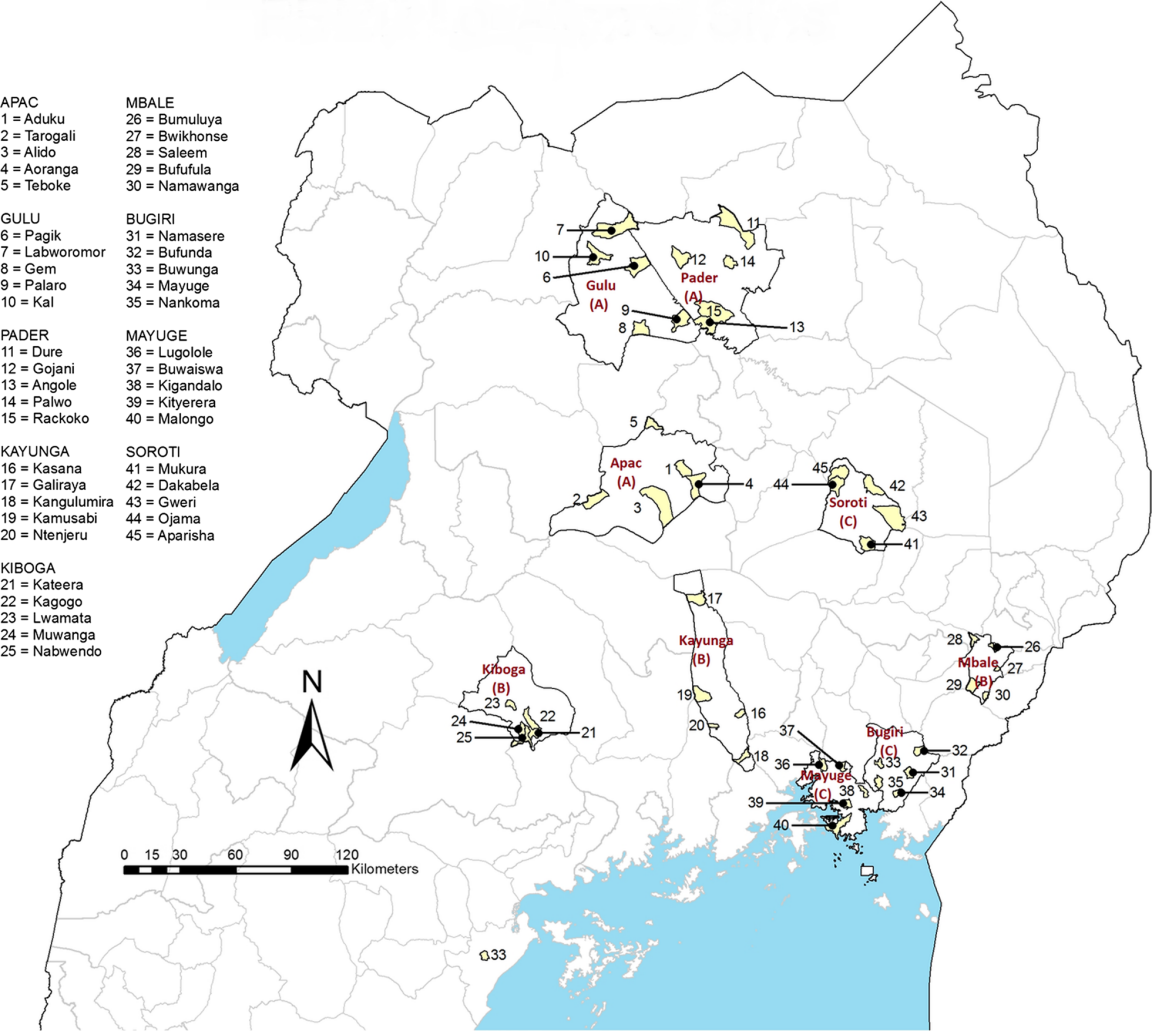
Study sites in Uganda. Group codes are shown in *brackets* under district names

In each district, five rural study sites were selected among health centre IIIs and IVs with long-term morbidity records (Fig. 1). Twelve rural households were selected randomly in each study site from the village around the health facility for household interviews, malariometric sample collection and entomological studies.

### Household and malariometric survey

A household interview was conducted during September–October 2012 to gather data on educational level of the household heads, household assets, knowledge of malaria, and mosquito nets owned. For each consenting resident, history of fever in the preceding 48 h and axillary temperature were recorded. Blood samples were taken and thin and thick blood smears prepared. Slides were stained with Giemsa and examined by two microscopists independently. Slides with discrepant results were re-examined by a third microscopist. Preparation of slides from study sites in Mayuge and Kaynuga districts did not meet the required standards for inclusion in the analysis.

### Entomological studies

The entomological survey was carried out during September–October 2012. The survey comprised two rounds of pyrethrum spray catch (PSC) in each of 12 randomly selected houses per site and insecticide susceptibility tests. Mosquitoes were identified to species complex level by morphological methods, and their blood digestion stages determined. Samples were then packed individually in Eppendorf Tubes^®^ and stored in bags containing silica gel, for subsequent molecular analysis.

Susceptibility of *An. gambiae* s.l. to deltamethrin, permethrin and bendiocarb was tested according to WHO guidelines available at the time of the survey [16]. Female mosquitoes were exposed to insecticide-impregnated papers in cylinder bioassays. Mortality rates were determined after a 24-h holding period. Mosquitoes used for testing were either collected as larvae and reared to adults (69% of samples) or collected using aspirators indoors as adults (31%). Females that were collected as larvae were one to four days old at the time of the tests. These mosquitoes were also packed for subsequent molecular analysis.

Molecular analysis of a sub-set of mosquito samples was carried out at Rothamsted Research, UK, using procedures developed by Bass et al. [17]. Genomic DNA was extracted using the Livak method [18]. *Anopheles gambiae* s.l. samples were analysed to determine whether samples were *An. gambiae* s.s. or *Anopheles arabiensis* [19, 20]. The samples were also analysed for *kdr* L1014S, *kdr* L1014F and acetylcholinesterase (*ace*-1 G119S) mutations [21, 22].

### Survey of agricultural insecticide use

A survey to understand agricultural insecticide use was conducted during April–May 2012. Data were collected from key informants based on their use of insecticides and interviews with heads of 12 randomly selected households in each study site. Data collected included: chemicals used, concentration, quantity used by year or growing season, frequency of application, source, target crops, and pests.

### Data analysis

EpiData version 3 was used for double-entry of data. Stata versions 12 and 13 (Statacorp) and Excel version 14 (Microsoft Corp) were used for data analysis. Sampling weights of households were computed from sampling frames within a multi-stage sampling design. The weights attached to each selected household reflected the probability that the household would be selected among all households in the village which were included in the sampling frame, given the probability of selection of the health centre within a district out of all eligible health centres in the district, and the probability of selection of each district within a group.

Mosquito nets owned by the households were classified as ITNs (which were all LLINs) or untreated nets. In statistical analysis involving historical exposure of the local vector populations to pyrethroids, nets received within three months prior to the survey were excluded.

Data from farmer interviews in the randomly selected households were used as it most accurately reflected the average insecticide use per study site. The amounts of active ingredients for each chemical and class of chemicals were calculated from use data and concentration of the formulations. The total amount of active ingredient per site was averaged over the total number of respondents in each site to obtain an average use by household.

Indoor resting density was estimated from PSC collection data, and calculated as the average number of females collected per house for each species complex. For resistance studies, the revised WHO guidelines were used to interpret mortality figures and determine resistance status [23]; test results were classified as: susceptible populations (≥98% mortality), suspected resistant populations (90 to <98%), or resistant populations (<90% mortality). Mortality rate results for *An. gambiae* complex species were re-calculated after molecular analysis of tested mosquitoes by pooling samples from various sites.

For regression analysis, *kdr* L1014S homozygous (RR) frequency (hereinafter referred to as *kdr* homozygosity) was used as the outcome variable to test the effect of various factors on the level of pyrethroid resistance using a logistic regression model. *Kdr* homozygosity was chosen instead of allele frequency as this mutation is known to be incompletely recessive and therefore functionally relevant only in the homozygous state. All *An. gambiae* s.s. mosquitoes from all study sites for which molecular analysis results were available for *kdr* L1014S were included. Each of the mosquitoes was classified as 0 (SS or RS) and 1 (RR). The following site-specific variables were entered in the regression model as independent variables:

- Spray status of the site (not sprayed; sprayed with bendiocarb in 2010 and 2011) (entered as categorical variable).
- Annual rainfall (mm) [24].
- Altitude (m).
- Collection type (collected as larvae; collected as adults indoors) (entered as categorical variable).
- Mean number of LLINs hanging per household during the survey, excluding nets obtained in the previous three months (LLIN use) (representing mean number of existing nets, and therefore pyrethroid pressure from ITNs in households).
- Number of LLINs known or estimated to be distributed in the site in the past 5 years.
- Total active ingredient (g) of carbamates used in agriculture during 2011 per household.
- Total active ingredient (g) of organophosphates used in agriculture during 2011 per household.
- Total active ingredient (g) of pyrethroids used in agriculture during 2011 per household.
- Malaria prevalence in children 2–9 years old during October–November 2012.

The survey setting in Stata was used to set study sites as primary sampling units and the three groups as strata. The *svy* command was then used together with the *logistic* command to select the best-fitting logistic regression model using stepwise backward regression.

## Results

### Insecticide pressure

Insecticide pressure was estimated from historical IRS data, ITN distribution data (historical data and LLINs found hanging during the survey), and information gathered on the use of agricultural insecticides at household level.

### IRS

Indoor residual spraying operations began in 2007 in Group A districts with pyrethroid insecticides primarily used in the first few years until the carbamate bendiocarb was introduced in 2010 [25]. Between 2007 and 2009, DDT was sprayed once in Apac, and lambda-cyhalothrin was sprayed three times each in Gulu and Pader districts. In 2010, alpha-cypermethrin was first used in all three districts before switching to bendiocarb in the same year. During the following year (2011), bendiocarb was sprayed twice in all three districts.

### ITNs

**Fig. 2:**
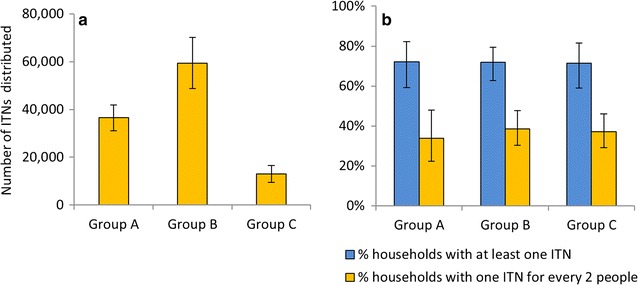
Historical and observed ITN coverage. **a** Estimated total number of ITNs distributed in the three groups of districts during 2001–2011; **b** Ownership of ITNs as determined from data gathered during household surveys in the study sites in the three groups of districts, September–October 2012. *Error bars* indicate 95% confidence intervals (CI)

The historical ITN distribution data gathered from various sources showed that sites in Group C received the least number of ITNs compared to sites in Groups A and B in the period 2001–2011 (Fig. 2a).

Study sites in Group B received the most nets. Most nets were LLINs (>95%) and 90% of nets were distributed after 2006. However, data collected during the survey showed that by the time the study started, no substantial differences were observed any more in ITN coverage between the three groups even when nets received within the previous three months were excluded (Fig. 2b).

### Agricultural insecticides

**Fig. 3:**
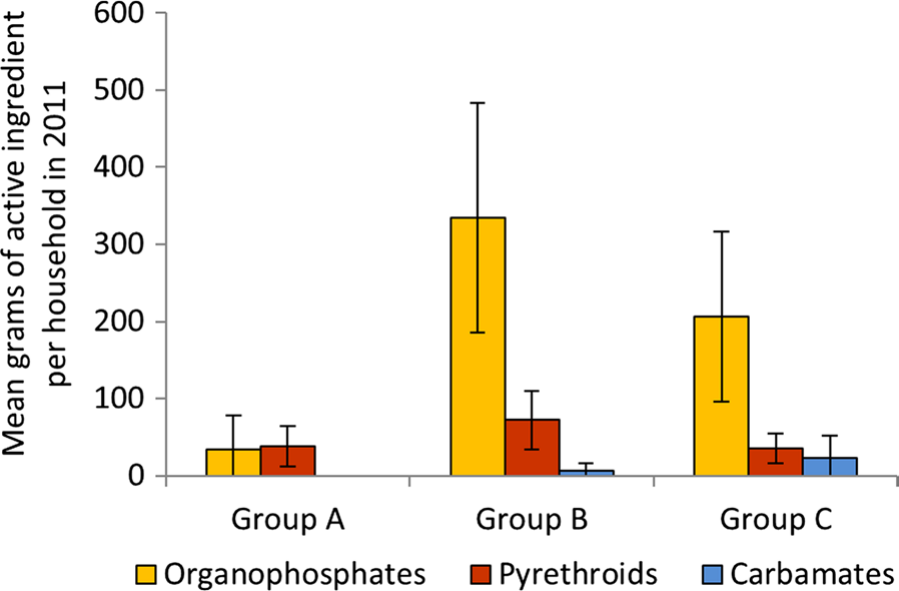
Average active ingredients (g) used per household in 2011 for agriculture. *Error bars* indicate 95% CI

Approximately half of the respondents indicated that they use insecticides for crops or cattle. There was a large variation between sites and districts. Agricultural insecticide use was most common in Soroti, while in Apac and Mayuge it was the lowest. Agricultural use data from sampled households showed that organophosphates were used at significantly greater amounts in Group B sites compared to sites in Groups A and C in 2011 (Fig. 3). The amount of pyrethroids used was similar between Groups A, B and C while the amount of carbamates used in Groups B and C was similar (carbamates were not used in Group A districts).

### Malaria prevalence

**Fig. 4:**
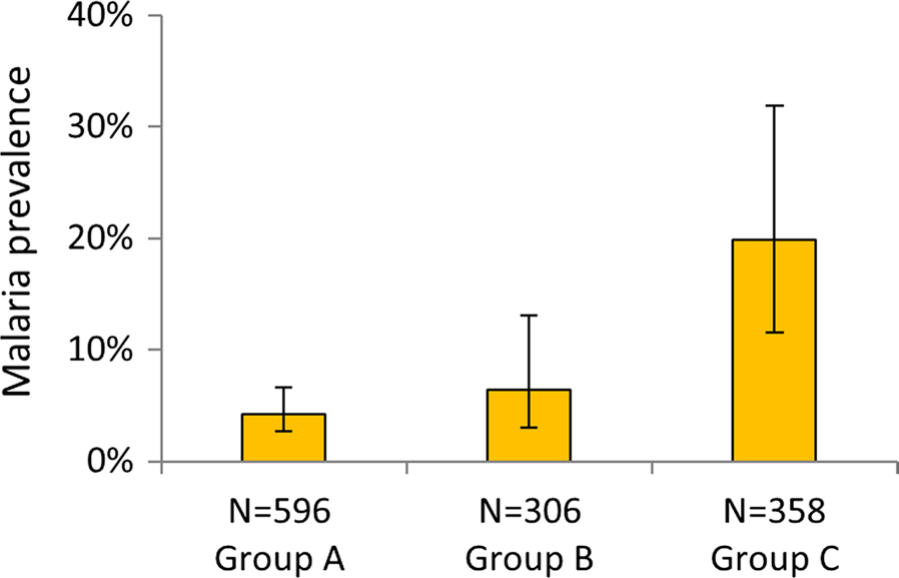
Prevalence of malaria infection in the study sites, September–October 2012. Kayunga and Mayuge data were not included. *Error bars* indicate 95% CI

Malaria prevalence in all age groups varied between the three groups of districts. Nearly all infections (99.2%) were due to *Plasmodium falciparum* and the rest due to *Plasmodium malariae*). Prevalence was lowest (4.3%) in the sprayed group of districts (Group A) and highest in Group C with the least historical coverage of LLINs (19.8%) (Fig. 4).

### Vector density

**Fig. 5:**
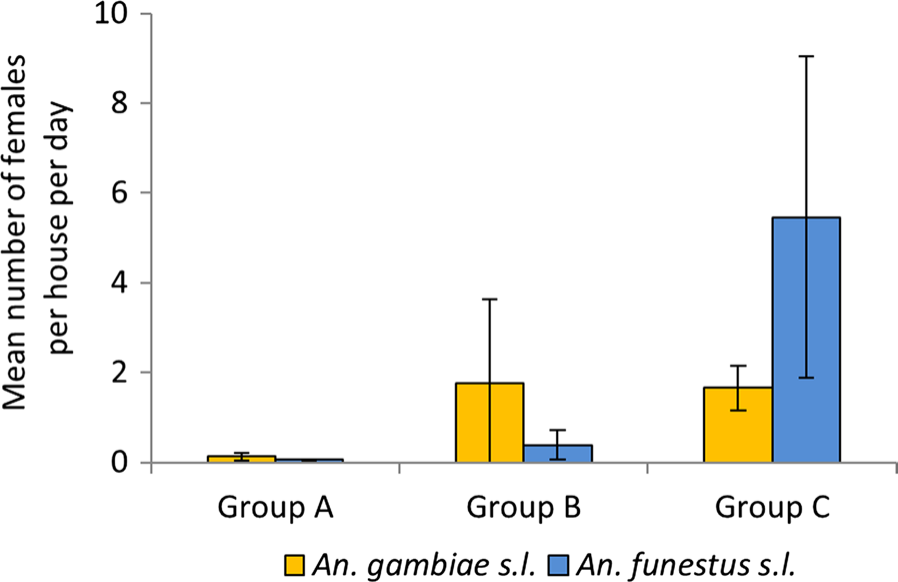
Indoor resting densities of *Anopheles gambiae* s.l. and *Anopheles funestus* s.l. Collected by PSC in Uganda, September–October 2012

*Anopheles funestus* s.l. and *An. gambiae* s.l. constituted 56 and 40% of the total anopheline mosquitoes collected, respectively. Significant differences were observed in indoor resting densities of *An. gambiae* s.l. and *An. funestus* s.l. between groups of districts. The lowest vector densities were observed in the sites under IRS in Group A (Fig. 5). *Anopheles funestus* s.l. was most common in Soroti district (in Group C). In Mbale (Group B) and Bugiri (Group C), both species groups were collected in similar proportions, while in all other districts *An. gambiae* s.l. was most common. *Anopheles funestus* s.s., *An. arabiensis*, and *An. gambiae* s.s. were sympatric in most districts. Apac, Gulu, Pader and Kiboga had overall very low vector densities, whereas Soroti had the highest.

### Insecticide resistance

**Fig. 6:**
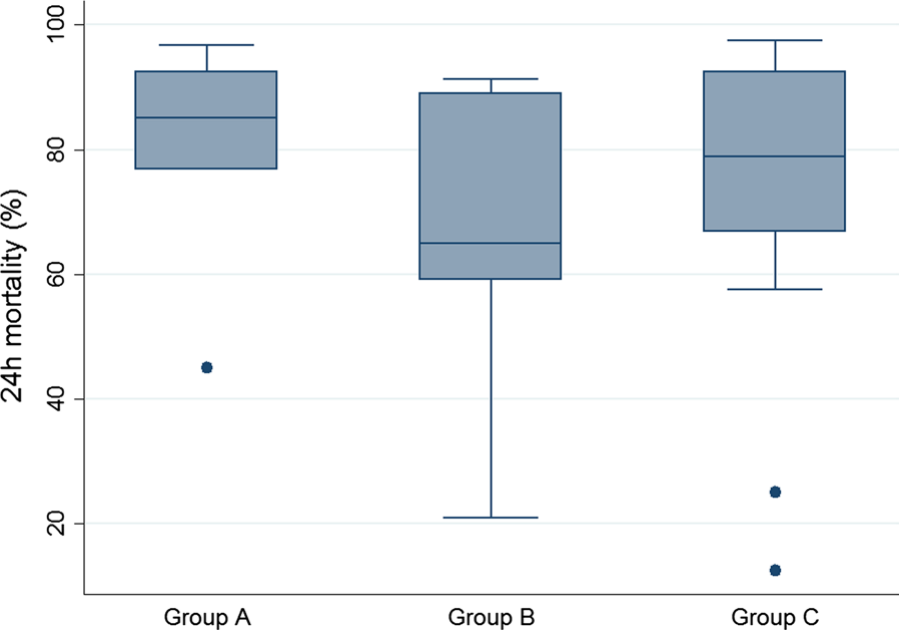
Mortality rates in *An. gambiae* s.l. tested with pyrethroids. The plots represent 24 h mortality rates from WHO tests with deltamethrin or permethrin using mostly or only *An. gambiae* s.l. mosquitoes collected as larvae (with n ≥ 40) for the three groups of districts

*Anopheles gambiae* s.l. was used for most tests. Bendiocarb resistance was not detected in any of the districts where IRS has been ongoing using this insecticide since 2010. In unsprayed districts, confirmed bendiocarb resistance was detected in Soroti only, although the sample size was below the recommended number. Mortality rates were calculated for tests with pyrethroids using mostly or only *An. gambiae* s.l. mosquitoes collected as larvae (with n ≥ 40) for the three groups of districts (Fig. 6). Significantly higher proportion of tests showed pyrethroid resistance or suspected resistance according to WHO classification in groups B and C districts (all 30 tests) compared with tests in group A districts (6 of 10 tests) (*p* = 0.002). Species-specific mortality rates were determined for *An. gambiae* s.s. and *An. arabiensis* post-molecular analysis (Table 1). Pyrethroid resistance was detected in *An. gambiae* s.s. in all districts except in Apac (Group A) where no resistance was detected and in Mayuge (Group C) where suspected resistance was detected with both deltamethrin and permethrin. Similar pyrethroid resistance levels were found in *An. arabiensis* except in Kayunga and Bugiri with suspected resistance to deltamethrin and permethrin, respectively.

**Table 1:**
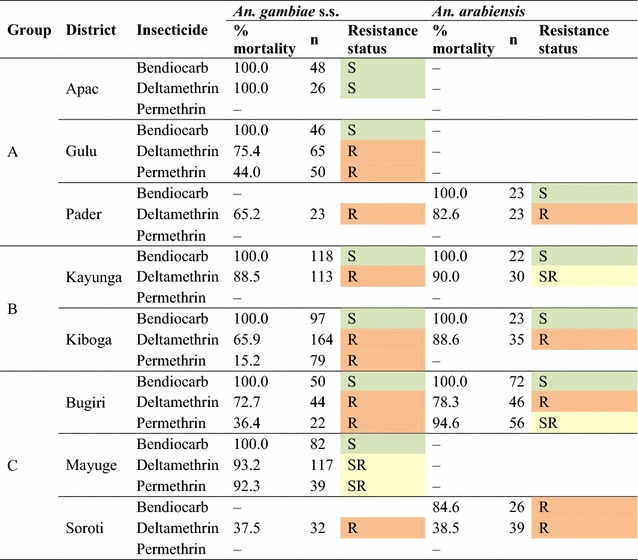
*Anopheles gambiae* s.s. and *Anopheles arabiensis* 24-h mortality results of insecticide susceptibility tests, 2012

Results presented were re-calculated after molecular analysis and based on pooled samples from various sites and only shown if the number of analysed mosquitoes was greater than 20 (S = susceptible, SR = suspected resistant, R = resistant). Tests were done in Mbale district (Group B) but results are not shown as insufficient samples were analysed for inclusion in the Table

Presence of *kdr* mutations was investigated in a sub-set of the *An. gambiae* s.l. sample. A total of 1910 *An. gambiae* s.s. samples and 638 *An. arabiensis* were successfully characterized for the L1014S mutation. Only a small number of mosquitoes from susceptibility tests in a few districts were analysed for the presence of *kdr* L1014F mutation as it was rare at the time, so the study focussed on *kdr* L1014S. Fifty-six *An. arabiensis* from Bugiri, Mayuge and Soroti districts were all homozygous susceptible for the L1014F mutation. Out of 21 *An. gambiae* s.s. samples from other districts, two were homozygous resistant and one heterozygous resistant, and the rest were susceptible. The *kdr* L1014S mutation was overall less common in *An. arabiensis* than *An. gambiae* s.s.; between the three groups, genotype frequencies varied between 31.2 and 35.7% in *An. arabiensis* compared to 85.7 and 94.1% in *An. gambiae* s.s. (Table 2). Hardy–Weinberg equilibrium tests showed deficiency of heterozygotes in unsprayed areas in *An. gambiae* s.s. mosquitoes collected as larvae (*p* < 0.001) and as adults (*p* < 0.001), while in sprayed areas the deficiency was observed only in mosquitoes collected as adults (*p* < 0.001) and not in mosquitoes collected as larvae (*p* = 0.104).

**Table 2:** *Kdr* L1014S genotype frequencies in *Anopheles gambiae* s.s. and *Anopheles arabiensis*, 2012

Group	District	*An. gambiae* s.s.				*An. arabiensis*			
		SS	RS	RR	% L1014S	SS	RS	RR	% L1014S
A	Apac	9	23	85	82.5	43	10	15	29.4
	Gulu	2	30	147	90.5	9	5	4	36.1
	Pader	7	7	26	73.8	30	4	13	31.9
	Sub-total	18	60	258	85.7	82	19	32	31.2
B	Kayunga	3	32	440	96.0	25	6	38	59.4
	Kiboga	6	34	263	92.4	66	4	5	9.3
	Mbale	3	10	53	87.9	38	9	17	33.6
	Sub-total	12	76	756	94.1	129	19	60	33.4
C	Buguri	9	5	151	93.0	112	12	55	34.1
	Mayuge	10	20	379	95.1	24	1	11	31.9
	Soroti	8	11	137	91.3	46	5	31	40.9
	Sub-total	27	36	667	93.8	182	18	97	35.7
Total		57	172	1681	92.5	393	56	189	34.0

*SS* homozygous susceptible, *RS* heterozygous, *RR* homozygous resistant

The acetylcholinesterase (*ace*-1 G119S) target mutation, which confers resistance against carbamate and organophosphate insecticides, was investigated for a sub-set of *An. gambiae* s.l. A total of 449 *An. gambiae* s.s. and 154 *An. arabiensis* were successfully characterized and all the samples were susceptible homozygotes. Although sample sizes were small for some districts, this result suggests that the *ace*-1 G119S mutation was rare in both species at the time of the survey.

### Effect of bendiocarb spraying on *kdr* homozygosity

**Fig. 7:**
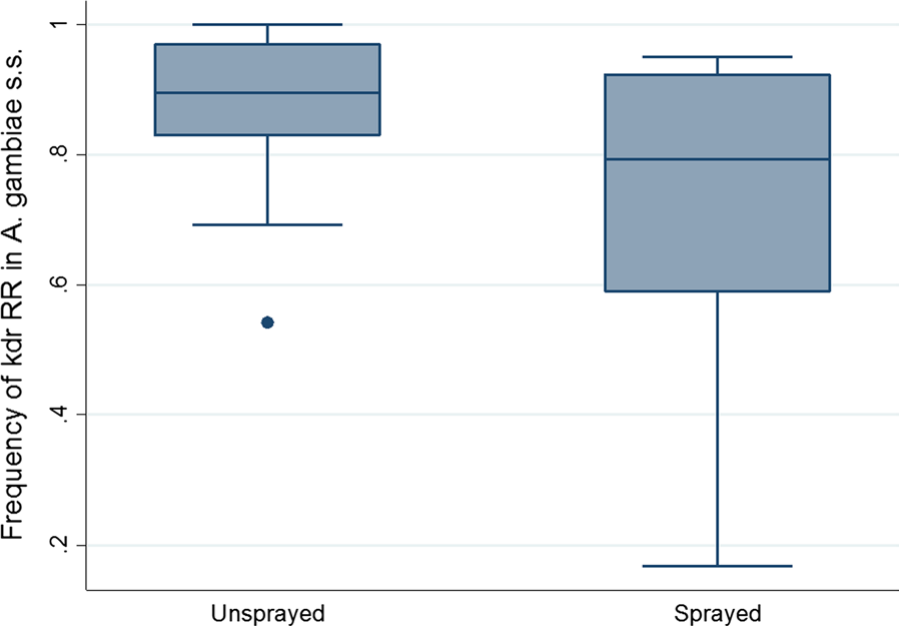
Frequency of *kdr* L1014S homozygotes in bendiocarb-sprayed and unsprayed areas in Uganda

Univariate analysis showed that the frequency of *kdr* L1014S homozygotes (*kdr* homozygosity) in *An. gambiae* s.s. in bendiocarb-sprayed sites was significantly less than the frequency in unsprayed sites (Fig. 7).

**Fig. 8:**
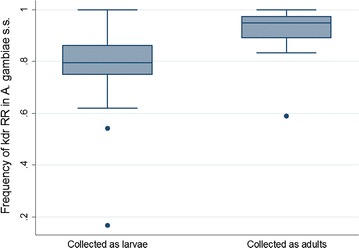
Frequency of *kdr* L1014S homozygotes in mosquitoes collected as larvae and as adult indoors

The overall *kdr* homozygosity in *An. gambiae* s.s. in sprayed sites was 73.4% (95% CI 58.8–84.2%) (n = 336), whereas in unsprayed sites it was significantly higher: 90.4% (95% CI 86.7–93.1%) (n = 1574) (*p* = 0.0016). *Kdr* homozygosity did not vary significantly within the sprayed group of districts (*p* = 0.119) and also within the unsprayed groups of districts (*p* = 0.372). Significantly higher levels of *kdr* homozygosity were observed in mosquitoes collected as adults than in mosquitoes collected as larvae (Fig. 8). The frequency was 91.6% (95% CI 85.3–95.4%) (n = 1110) in mosquitoes collected as adults indoors, whereas it was 81.0% (95% CI 74.8–85.9%) (n = 800) in mosquitoes collected as larvae (*p* = 0.0109).

Spray status, collection type and the interaction of LLIN use and annual rainfall had statistically significant effects (Table 3). *Anopheles gambiae* s.s. collected from areas sprayed with bendiocarb had significantly lower *kdr* homozygosity compared to mosquitoes collected from unsprayed areas. The odds ratio for sprayed sites was 0.328 (95% CI 0.173–0.623), after controlling for potential confounding variables.

**Table 3:** Results of logistic regression analysis of *kdr* L1014S homozygosity in *Anopheles gambiae* s.s. in Uganda

Variable	OR	SE	*t*	*p*	[95% CI]
Spray status					
Not sprayed	1				
Sprayed with bendiocarb	0.328	0.103	−3.55	0.001	[0.173, 0.623]
Collection type					
Collected as larvae	1				
Collected as adults indoors	2.847	0.543	5.49	<0.001	[1.928, 4.202]
Annual rainfall (mm)	0.987	0.003	−4.45	0.010	[0.981, 0.993]
LLINs found hanging (LLIN use)	1.53 × 10^−5^	6.21 × 10^−5^	−2.74	0.001	[3.89 × 10^−9^, 0.060]
Annual rainfall × LLINs found hanging	1.008	0.003	−2.69	0.012	[1.002, 1.015]

Number of strata = 3; study sites included = 33; number of mosquitoes = 1910; design degrees of freedom = 30; F(5,26) = 37.5; *p* < 0.001

The results of the regression analysis showed that mosquitoes collected as adults from indoor collections had significantly higher frequency of *kdr* homozygotes than mosquitoes collected as larvae. *Kdr* homozygosity in indoor-collected adults was 2.85 times that of mosquitoes collected as larvae. The effect of the number of LLINs found hanging on *kdr* homozygosity was significantly modified by annual rainfall. In areas receiving high rainfall, the number of LLINs was associated with increased *kdr* homozygosity and this association weakened as rainfall decreased (average annual rainfall ranged from 1170 to 1464 mm with a mean of 1268 mm [26]). Altitude, LLINs estimated to have been distributed in the past, amount of household agricultural insecticides used and prevalence of malaria among children 2–9 years old were not associated with *kdr* homozygosity.

## Discussion

These data show that *An. gambiae* s.l. populations are resistant to pyrethroids in most of the study sites examined and also probably to bendiocarb in at least one site in eastern Uganda. The results confirm previous findings [27, 28]. In this study, pyrethroid resistance was not detected in one of the sprayed districts (Apac). Conflicting results have been reported from Apac in the past: while a study in 2011 showed widespread resistance against all pyrethroids tested [28], full susceptibility against deltamethrin was reported from the district in 2009 [27]. Unpublished Malaria Consortium data also indicated some level of resistance after the present study. In the other two sprayed districts, Gulu and Pader, resistance to deltamethrin and permethrin was detected.

The L1014S *kdr* mutation was identified at high frequency in *An. gambiae* s.s. and at moderate frequency in *An. arabiensis* in all districts. While this *kdr* variant was common (although it was relatively less frequent in sprayed areas), L1014F *kdr* mutation was rare in *An. gambiae* s.s. and absent in *An. arabiensis*, in line with results from other studies in Uganda [29–31]. Moreover, the *ace*-1 G119S mutation was absent from *An. gambiae* s.s. and *An. arabiensis*, similar to findings reported from another study in eastern Uganda in 2008 [31].

Genotyping data suggested that bendiocarb has possibly led to reduced *kdr* frequency despite the high selection pressure produced by net coverage and usage in the sprayed sites in Group A. Although bendiocarb spraying did not result in elimination of the *kdr* resistant genotypes, there is an association which merits further study. IRS of insecticides with different mode of action to pyrethroids may be an effective resistance management strategy.

The increased *kdr* homozygosity in mosquitoes collected indoors as adults compared to those collected as larvae might be explained by exposure to pyrethroid-treated nets prior to collection with adult mosquitoes of SS or RS genotypes killed by exposure to LLINs (as *kdr* is an incompletely recessive trait in mosquitoes). A similar phenomenon has been observed in a study in western Kenya, in which mosquitoes reared from females collected inside nets had lower susceptibility to pyrethroids, compared to those from larval collections [32].

The effect of rainfall on *kdr* homozygosity was not large but was significant. Average annual rainfall varied little between the sites. The effect of the number of LLINs found hanging (which could represent to number of LLINs present in the households) on *kdr* homozygosity was found to be significantly modified by rainfall. In areas receiving high rainfall, LLIN use was associated with increased *kdr* homozygosity and this association weakened as the amount of annual rainfall decreased.

Although the intensive use of pyrethroids in agriculture has been reported to have contributed to the appearance of resistance in some areas of Africa, the massive scale-up of LLINs and IRS for malaria control is thought to be the main driver of the increasing resistance problems reported [15]. Selection pressure from household-level use of insecticides in small-scale agriculture was not significant for pyrethroids, carbamates and organophosphate insecticides in this study. However, detection of bendiocarb resistance in Soroti district might have resulted from carbamates used in agriculture as there had been no IRS in the district at the time of the study. A number of studies found a positive association between resistance levels in vectors and insecticide use in farming [33–35]. However, the intensity and frequency of use in agricultural areas reported in the studies was likely to be far greater than the small-scale household use investigated in the present study. The variation between sites in terms of intensity of agricultural insecticide use in the present study may not have been large enough to show the effect on *kdr* frequencies. LLIN coverage is likely to be the driving force in the study areas.

The effect of the massive increase in the use of the core malaria vector control interventions (LLINs and IRS) in Africa on resistance against pyrethroids is well established [36]. While the present study showed that groups of districts where, historically, relatively low number of LLINs were distributed through campaigns (Group C) had the highest vector density and malaria prevalence, it also indicated that the high ownership of LLINs found during the survey led to increased *kdr* homozygosity especially in sites receiving high rainfall. The results of the regression analysis suggest that the presumably higher mosquito densities in wet areas could increase the frequency of exposure to ITNs, as high vector density would encourage more consistent use of LLINs due to biting nuisance than in relatively drier areas, thus increasing exposure of local vectors to pyrethroids.

The statistical analysis in this study revealed that, after controlling for the potential effects of insecticide pressure and level of exposure to insecticides from LLINs present in households, and the effect of mosquito collection methods used, the effect of bendiocarb spraying on *kdr* homozygous frequency was highly significant. The global recommendation has been to use non-pyrethroid insecticides for IRS in areas where LLINs are common [37]. Evidence of a reversal of resistance to a susceptible population following the removal of selection pressure has been reported in some settings, while in others the rate of reversal has been very slow [38]. The results of the regression analysis in the present study indicated that bendiocarb spraying could potentially reduce *kdr* homozygosity by 67.2% (95% CI 37.7–82.7%) with the selection pressure of pyrethroid-treated nets included in the model, but may not be able to eliminate the mutation once a high frequency has been attained. *Kdr* homozygosity may not always be directly associated with elevated vector survival or sporozoite infection rates [26]. In this study metabolic resistance was not investigated; yet it is more likely to be associated with control failure than *kdr* frequencies [2]. *Kdr*-L1014S frequencies and phenotypic resistance levels did not match well (e.g. WHO test survivors did not always have *kdr* genotypes), which suggests that at least one other mechanism was responsible for the resistance phenotype observed. Resistance mediated by cytochrome P450 monooxygenases may exist in the vector populations. *Kdr* homozygosity is not necessarily equivalent to phenotypic resistance and that the presence of other mechanisms of resistance could have confounded the results of the analysis. Furthermore, there are some variations in phenotypic resistance between the sprayed districts although statistical tests did not show a significant difference with *An. gambiae* s.l. However, low levels of pyrethroid resistance were observed in Apac. It is possible that the observed effects may have been driven by data from a limited number of sites that could be outliers. Nevertheless, the study suggests that using a carbamate for IRS in areas with high levels of pyrethroid resistance could have a substantial impact on vector density and malaria transmission and may reduce *kdr* frequencies in *An. gambiae* s.s.
